# Markov Chain Modeling of HIV, Tuberculosis, and Hepatitis B Transmission in Ghana

**DOI:** 10.1155/2019/9362492

**Published:** 2019-11-20

**Authors:** Clement Twumasi, Louis Asiedu, Ezekiel N. N. Nortey

**Affiliations:** ^1^School of Mathematics, Cardiff University, Cardiff, UK; ^2^Department of Statistics & Actuarial Science, School of Physical and Mathematical Sciences, University of Ghana, Legon, Accra, Ghana

## Abstract

Several mathematical and standard epidemiological models have been proposed in studying infectious disease dynamics. These models help to understand the spread of disease infections. However, most of these models are not able to estimate other relevant disease metrics such as probability of first infection and recovery as well as the expected time to infection and recovery for both susceptible and infected individuals. That is, most of the standard epidemiological models used in estimating transition probabilities (TPs) are not able to generalize the transition estimates of disease outcomes at discrete time steps for future predictions. This paper seeks to address the aforementioned problems through a discrete-time Markov chain model. Secondary datasets from cohort studies were collected on HIV, tuberculosis (TB), and hepatitis B (HB) cases from a regional hospital in Ghana. The Markov chain model revealed that hepatitis B was more infectious over time than tuberculosis and HIV even though the probability of first infection of these diseases was relatively low within the study population. However, individuals infected with HIV had comparatively lower life expectancies than those infected with tuberculosis and hepatitis B. Discrete-time Markov chain technique is recommended as viable for modeling disease dynamics in Ghana.

## 1. Introduction

Tuberculosis (TB) is predominant among patients with human immunodeficiency virus (HIV) according to the WHO [1] report. Thus, TB is considered as the main cause of mortality among individuals susceptible to HIV [2]. Thus, the combined effect of the two diseases is regarded as extremely fatal as compared to the individual or marginal effect of each of the underlying diseases on the individual. Additionally, WHO [3] confirmed that most HIV patients contract tuberculosis as the first evidence of AIDS, with about 33.3% of 38.6 million HIV-positive patients worldwide, also infected with tuberculosis, and are all at risk of contracting fully TB disease. Among other possible means of HBV infection spread, the dominant modes of transmission for the high prevalence of the disease infection in the community under study (Kumasi, Ashanti region) and its surroundings are usually through transfusion of infected blood, unprotected sexual activities, use of unsterilized blades or barbering equipment, and mother-to-child transmission during delivery [4]. On the other hand, HIV spread is associated with sexual risk behavior among HIV-positive individuals, which consequently put uninfected persons at risk of HIV infection.

According to [5], out of 36 million HIV-infected individuals, approximately 4 million were found to have chronic HBV.

The immune system which is the body's natural defense system against pathogens, infections, and illnesses is made of some special cells known as the CD4 cells. These underlying cells are white blood cells that fight against various forms of infections in the human system and thus provide an overview of the performance of the immune system. Hence, the count of CD4 cells in an individual at any time determines the strength of his or her level of immunity. However, the counts of CD4 cells are mostly affected by viral and bacterial infections. This explains why the combined effects of any two of these underlying diseases (HIV, TB, and HB) on an individual are extremely dangerous [6]. Consequently, these diseases under consideration must be viewed as a public health concern in Ghana and the world at large. Among adults and adolescents in Ghana, the first-line ART regimens prescribed comprises of a nonthymine nucleoside reverse transcriptase inhibitors (tenofovir + emtricitabine or tenofovir + lamivudine) and one nonnucleoside reverse transcriptase inhibitor normally efavirenz [7].

Several mathematical and standard epidemiological models have been proposed in studying infectious disease dynamics. These models help us to understand the spread of disease infections. However, most of these models are not able to estimate other relevant disease metrics such as probability of first infection and recovery as well as the expected time to infection and recovery for both susceptible and infected individuals. Most of the standard epidemiological models used in estimating transition probabilities (TPs) are not able to generalize the transition estimates of disease outcomes at discrete time steps for future predictions. This paper seeks to address the aforementioned problems by adopting a discrete-time Markov chain model as proposed in [8]. In this study, the *n*th-step transition probability matrices for each disease are determined using the first-order Markov chain model. The findings would help policy makers to ascertain which of the diseases under study are most infectious and deadly so that measures could be put in place to reduce or minimize its prevalence.

## 2. Materials and Methods

### 2.1. Source of Data

The data used for the study were collected from a regional hospital in Ghana since it serves as a major referral center. Data were obtained from periodic follow-ups of HIV, TB, and hepatitis B patients from January 2016 to December 2016. Each patient was followed for a year irrespective of their entry times in the course of the year. Patients with TB relapse or reinfection as well as patients with confirmed coinfections or other medical complications (and lost to follow-up) were excluded or withdrawn for medical attention. Among subjects susceptible (uninfected) to these diseases after medical tests, counts of those who were found infected and dead (or immune to the disease possibly) by the end of the study period (till the last eligible subject was followed for a year) were recorded. Similarly, among subjects infected in the course of the study (after medical screening for each disease), counts of those who recovered (TB only), remained infected, and died were also recorded.

### 2.2. S-I-R Modeling by Markov Chain Process

#### 2.2.1. Model Development

Consider three discrete states: susceptible (state 0), infected (state 1), and removed (state 2) states. If (*X*_*i*_, *i*=0,1,2) represent the number of individuals at any state from the underlying diseases at any time *t*, then clearly, *X*_*i*_ is a stochastic process with states 0, 1, and 2.

Thus, the first-order time-homogeneous Markov dependency can statistically be modeled as

(1) $$\begin{array}{c} P\left(X_{n}=i_{n}\;\left|\;\right.X_{n-1}=i_{n-1},\ldots ,X_{1}=i_{1},X_{0}=i_{0}\right)\\ =P\left(X_{n}=i_{n}\;\left|\;\right.X_{n-1}=i_{n-1}\right). \end{array}$$

Then, the transition probability (*P*_*ij*_) for *i*, *j*=0,1,2 is denoted in matrix form as

(2) $$\begin{array}{c} \left(P_{ij}\right)=\left(\begin{array}{ccc} P_{00} & P_{01} & P_{02}\\ P_{10} & P_{11} & P_{12}\\ 0 & 0 & 1 \end{array}\right), \end{array}$$

where

(3) $$\begin{array}{c} \overset{2}{\underset{j=0}{\sum }}P_{ij}=1,\;i=0,1,2. \end{array}$$

#### 2.2.2. Definition of States in the S-I-R Model

  Susceptible state (S): it comprised of individuals who have not been exposed before and individuals who have recovered from an infection.
  Infectious state (I): it comprised of infected individuals and carriers of the disease.
  Removed state (R): it comprised of individuals who either died from the disease or were immune after recovery from the diseases (HIV excluded) in the course of the study period.

#### 2.2.3. Parameters of the Markov Chain (Probabilities of Transition)

 
*P*_*ii*_: probability of remaining in a state *i*.
 
*P*_*ij*_: transition probability from state *i* to state *j*, *i* ≠ *j*.

**Remark 2 s.**

The parameter *P*_01_ is mostly referred to in the literature as discrete time force of infection. Also, the elements *P*_02_ and *P*_12_ signify mortality for uninfected and infected individuals, respectively, while *P*_10_ is the recovery or defection probability [9]. Death is an absorbing state since the probability of becoming susceptible or infected is zero. The time step unit to ensure the transition from one state to another is measured on a yearly basis.

### 2.3. Model Assumptions

  The current state of an individual is dependent only on the state of the individuals at the previous time step.
  No individual at the removed state can be susceptible or infected.
  Transitioning probabilities are independent of time and remain constant over time or the study period.
  Successive transitions or relapse confirmed coinfections of the diseases or other medical complications were not taken into consideration or did not meet the eligibility criteria of the study.
  The removed state comprised of subjects who either died from the disease or found to be immune after recovery.
  The only assumption required regarding losses and withdrawals is that they have the same future experience as those remaining under observation.

### 2.4. Estimating Transition Probabilities

The maximum-likelihood estimation (MLE) was used to estimate the transition probabilities for each disease with their respective standard errors.  Table 1 shows the number of individuals during the study period at any state (S-I-R) for each cohort study for the three diseases (HIV, TB, and HB).

**Remark 1 s.**

The number of infected individuals who recovered at the end of the second stage or study period of the cohort studies for both HIV and HB would be zero since HIV and chronic hepatitis B are not curable as opposed to TB, but can be treated. There exists a finite treatment, especially for many cases of hepatitis B.

The transition events are independent of one another (as defined by the Markov principle); the likelihood of the transition probability, *P*_*ij*_, follows a binomial model:

(4) $$\begin{array}{c} L\left(P_{ij}\;\left|\;\right.N,x\right)=\left(\begin{array}{c} N_{i}\\ x_{ij} \end{array}\right)P_{ij}^{x_{ij}}{\left(1-P_{ij}\right)}^{N_{i}-x_{ij}}, \end{array}$$

where *N*_*ij*_ is the number of observed transition that starts from state *i* to *j* and

(5) $$\begin{array}{c} \underset{j}{\sum }P_{ij}=1. \end{array}$$

From equation (4) and the assumption of constant transition probabilities over the period, the transition probability matrix is estimated as a multinomial distribution given as

(6) $$\begin{array}{c} {\hat{P}}_{ij}=\frac{x_{ij}}{\sum_{j}x_{ij}}=\frac{x_{ij}}{N_{i}}, \end{array}$$

for *i*, *j*=0,1, with standard errors from the sampling distribution of the ML estimate given as

(7) $$\begin{array}{c} \hat{s}.e\left(P_{ij}\right)=\sqrt{\frac{{\hat{p}}_{ij}\left(1-{\hat{p}}_{ij}\right)}{N_{i}}}. \end{array}$$

### 2.5. Estimating Disease Metrics

The probability that a susceptible individual becomes infected for the first time between *m* − 1 and *m* time steps for states *i*, *j*=0,1 from the transition probability matrix (S-I-R) is given as

(8) $$\begin{array}{c} f_{01}^{\left(m\right)}=P\left(X_{n+m}=1,X_{n+m-1}=0,\ldots ,X_{n+1}=0\;\left|\;\right.X_{n}=0\right)\\ =P_{00}^{m-1}P_{01}. \end{array}$$

Similarly, the probability that an infected individual first recovers between *m* − 1 and *m* time steps is given as

(9) $$\begin{array}{c} f_{10}^{\left(m\right)}=P\left(X_{n+m}=0,X_{n+m-1}=1,\ldots ,X_{n+1}=1\;\left|\;\right.X_{n}=1\right)\\ =P_{11}^{m-1}P_{10}. \end{array}$$

The expected time to infection and recovery has a closed-form solution which is computed as

(10) $$\begin{array}{c} E\left(\tau_{ij}^{1}\right)=\frac{\sum_{m=1}^{\infty }mf_{ij}^{m}}{P_{\text{r}}\left(i⟶j\right)}\\ =\frac{1}{1-P_{ii}}, \end{array}$$

for *i*, *j*=0,1, *i* ≠ *j*, where the numerator, ∑*mf*_*ij*_^*m*^, is the expected value of first passage time from state *i* to state *j* and the denominator

(11) $$\begin{array}{c} P_{\text{r}}\left(i⟶j\right)=\frac{P_{ij}}{1-P_{ii}} \end{array}$$

is the overall probability or lifetime probability of transitioning from state *i* to state *j* [8].

The life expectancies (*W*_*i*_, *i*=0,1) for susceptible and infected individuals can also be estimated using the following equation:

(12) $$\begin{array}{c} W={\left(I-Q\right)}^{-1}\;\times \;\left(\begin{array}{c} 1\\ 1 \end{array}\right), \end{array}$$

where *I* is a 2 × 2 identity matrix and

(13) $$\begin{array}{c} Q\;=\;\left(\begin{array}{cc} P_{00} & P_{01}\\ P_{10} & P_{11} \end{array}\right). \end{array}$$

### 2.6. Estimating *P*^*n*^ Transition Matrix

The method for estimating the *n*th-step transition probability matrices for each disease uses the eigenvalue and eigenvector approach as proposed by Bhat [10]. The *P*_*ij*_^*n*^, *i*, *j*=0,1,2 transition probability matrix was estimated for each disease using a decomposition method that requires eigenvalues and their corresponding eigenvectors. Hence, it can be estimated using the decomposition below:

(14) $$\begin{array}{c} P^{n}=Q\Lambda^{n}Q^{-1}, \end{array}$$

where *Q* is 3 × 3 nonsingular matrix (*X*_0_, *X*_1_, *X*_2_) and *X*_*j*_, (*j*=0,1,2) is the right eigenvectors corresponding to the eigenvalues *λ*_*j*_(*j*=0,1,2). Thus,

(15) $$\begin{array}{c} PX_{j}=\lambda_{j}X_{j}, \end{array}$$

(16) $$\begin{array}{c} \Lambda^{n}=\left(\begin{array}{ccc} \lambda_{0}^{n} & 0 & 0\\ 0 & \lambda_{1}^{n} & 0\\ 0 & 0 & \lambda_{2}^{n} \end{array}\right). \end{array}$$

## 3. Results and Discussion

### 3.1. Markov Chain S-I-R Model

Suppose that the discrete states of the Markov chain model for the diseases (HIV, TB, and HB) are susceptible (state 0), infected (state 1), and removed or dead (state 2) states. Let (*X*_*i*_, *i*=0,1,2) represent the number of individuals at any state from the underlying diseases at any time *t* which satisfies the first-order time-homogeneous Markov dependency from equation (1). Clearly, *X*_*i*_ satisfies the Markov chain model with state space *S*={0,1,2}.

Tables 2–4 show the number of individuals within the study population at states 0, 1, and 2 for HIV, TB, and HB, respectively.

### 3.2. Estimating the Transition Probabilities for Each Disease

The maximum likelihood was used to estimate the transition probability matrix for each disease. Tables 5–7 show the estimates and confidence intervals of the transition probabilities for HIV, TB, and HB, respectively.

The transition probability matrices for the diseases are, respectively, presented as 
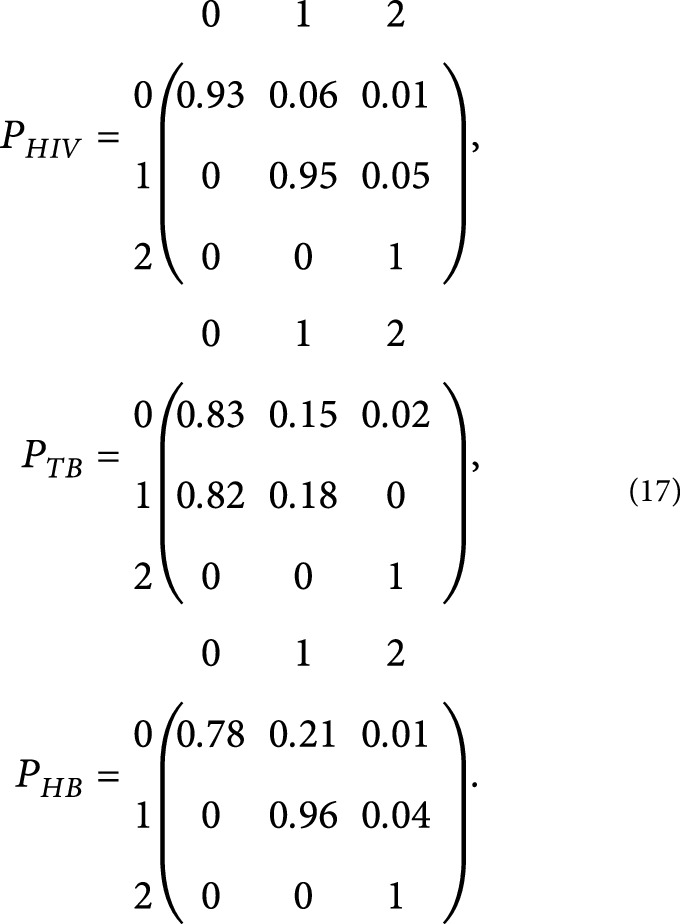


### 3.3. Classification of Model States Using Graph Algorithm

**Figure 1:**
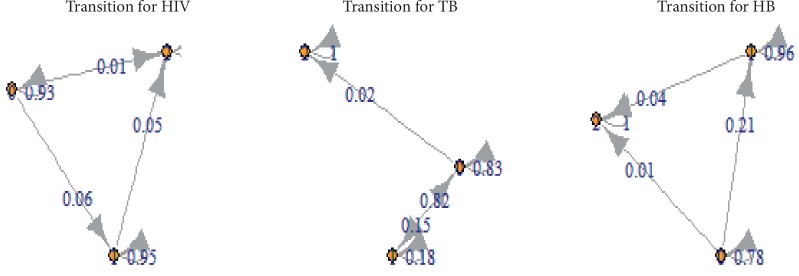
Directed multigraph of transitioning from one state to another.

A graph algorithm was used to represent the transition probabilities for each disease so as to easily classify the states into recurrent, transient, or absorbing. From Figure 1, it can be concluded that states {0} and {1} are both transient states for transitions with respect to HIV and hepatitis B. This is because, upon an individual leaving those states, there is no positive probability of returning to its original state. However, state {0, 1} is a transient class for transitions regarding tuberculosis since the two states have the same equivalence class as well as communicates, but, upon an individual leaving that class, there is no positive probability of returning to that class. State {2} which is also known as the removed state is an absorbing state for any disease transitions since an individual at this state remains dead with a certain probability of one. Therefore, it can be inferred from the state classifications that the Markov chain is not ergodic. That is, even though the chain is aperiodic, it is not irreducible since all the states do not belong to the same equivalence class. Figure 1 shows a graphical representation of the transition probabilities, respectively, for each disease.

### 3.4. Estimating Disease Metrics

#### 3.4.1. Probability of First Transition

**Figure 2:**
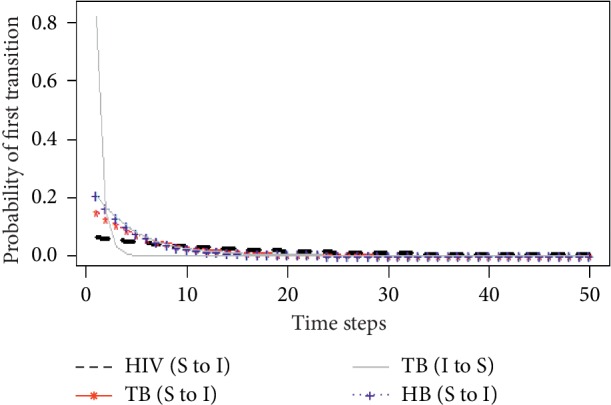
Probability of first infection and recovery.

The probability that a susceptible individual becomes first infected was estimated for all the underlying diseases using equation (8). In addition, the probability that an infected individual first becomes susceptible or recovers was estimated for only tuberculosis using equation (9) since HIV and hepatitis B have no cases of recovery from infection.  Figure 2 shows a plot of first transition probabilities across various time steps from 1 to 50.

From Figure 2, it can be observed that the probability of first infection of the three underlying diseases within the study population was relatively low (below 0.20). This could be due to the interventions (or medications) administered to the study population to control the spread of these diseases [11]. Moreover, it can also be inferred that, before the 10th time step (in 10 years after the study period), the probability of first hepatitis B infection by a susceptible individual was relatively higher, followed by tuberculosis infection and then HIV infection. On the contrary, the probability of first infection after the 10th time step was comparatively higher among HIV patients as opposed to both TB and HB patients. This finding is consistent with that obtained in [12], where the majority of people exposed to HIV first get infectious after 10 years of exposure without treatment. Also, the probability of first recovery from tuberculosis was very high at lower time steps but declined sharply across increasing time steps. Thus, it simply suggests that if a patient is diagnosed of TB at the early or latent stage, lasting control measures can be put in place to first recover from the disease. This is because most people with active TB after receiving proper treatment for at least 2 weeks are no longer contagious. However, if a TB patient is infected for a longer period, then there is a relatively very small probability of recovering from the disease in some future time steps.

### 3.5. Probability of Infection at Any Time Period

**Figure 3:**
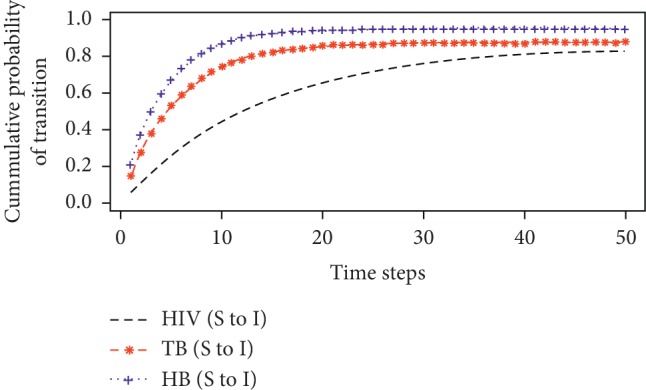
Probability of infection over the years for infected individuals.

The probability of infection at any time from the three underlying diseases was estimated using the cumulative sum of the probabilities of first infection of susceptible individuals.  Figure 3 is a plot of the cumulative transition probabilities over time.

The estimation of the probability of infection from the first transition probabilities as shown in Figure 3 revealed that hepatitis B appeared to be more infectious over time within the study population, followed by tuberculosis and HIV infections, respectively. This finding agrees with outcomes from other health surveys. WHO [12] from their studies over the years also found that even though the mode of transmission of HIV and HB are similar, hepatitis B is about 50 to 100 times more infectious than HIV. This is because HB virus unlike HIV can even live outside the host for at least a week and still cause infection in a susceptible individual as well as have a relatively higher rate of viral incubation. In addition, blood levels associated with the HBV are relatively higher than for HIV, thereby causing this virus to be easily transmitted in situations such as child delivery (from mother to child) and even through body fluids, which cannot happen in the case of HIV transmission. On the other hand, hepatitis B was also found to be more contagious than tuberculosis since the probability of HB infection at any time was relatively higher than TB spread. Bacterial infections such as TB are mostly cured by antibiotic drugs which are ineffective against viral infections such that their infection dynamics can even be controlled unlike hepatitis B at any stage of exposure. However, HIV seems less infectious after first infection comparatively and this could be due to the fact that the virus first attacks the immune system of individuals which usually takes about 5 to 10 years to get it fully compromised even when left untreated [12]. Thus, individuals with very strong immune system develop the infection at a relatively lower rate as opposed to TB and HB infections which also affects the immune system. It was also revealed that the probability of infection of these three diseases increases with increasing time period. This further suggests that HIV, TB, and HB get very infectious with increasing time if left unattended. This accounts for the deadliness of these three diseases due to its high rate of infection over the years. It also confirms why the combined effect of any two of these diseases on an individual can be extremely deadly as compared to the individual effects on persons suffering from either HIV, TB, or HB.

### 3.6. Other Estimated Disease Metrics

#### 3.6.1. Overall Probability of Infection and Recovery

Other disease metrics such as the overall probability of infection and recovery were estimated using equation (11). The overall probability of infection for HIV, TB, and HB was found to be approximately 0.86, 0.88, and 0.95, respectively. These findings are consistent with results obtained from the cumulative sums of the first infection probabilities as presented by Figure 3, where HB was more infectious over time as opposed to TB and HIV. Hence, it can be inferred that hepatitis B was the most infectious disease during the study period at the regional hospital. The study also found that it is certain to cure tuberculosis as opposed to HIV and HB which are incurable at the infectious or chronic stage.

#### 3.6.2. Assessing the Statistical Significance of the Overall Probability of Infection

The statistical significance of the estimate of the overall probability of infection for each disease from the Markov model was measured using bootstrap interval estimation technique at 1% level of significance. Hence, it can be concluded with 99% level of confidence that the overall probability of infection for each disease would fall in the estimated confidence intervals as summarized in Table 8.

### 3.7. Expected Time to Infection and Recovery

Also, the expected time to infection and recovery was estimated from equation (10) for each disease. The expected time to infection by a susceptible individual was found to be 14.29 years, 5.88 years, and 4.55 years for HIV, TB, and HB infections, respectively, within the cohort considered. That is, an individual exposed to HIV will on the average start experiencing the infection after approximately 14.29 years even though the length of time can vary widely between individuals. This finding affirms that obtained in [12], where individuals exposed to HIV fully developed the signs and symptoms of the infection usually between 10 and 15 years. On the other hand, the average time at which an individual exposed to TB would become fully infectious if left untreated was estimated as 5.88 years after exposure, whereas that of HB was estimated as 4.55 years. Hence, it can be inferred that hepatitis B infection develops faster on the average as compared to HIV and tuberculosis infections over time.

On the contrary, TB was found to have an expected recovery time of 1.22 years, whereas the expected time at which the symptoms and signs of HIV and HB would have been diminished was estimated as 20 years and 25 years, respectively. However, the expected to recover from HIV and hepatitis B as defined for this study coincided with the estimate of their respective life expectancies for infected individuals using the Markov chain model. Hence, it can be concluded that the life expectancy of an infected individual with respect to HIV and HB is indeed the period beyond which an infected individual recovers or dies.

### 3.8. Life Expectancy for Healthy and Infected Individuals

Life expectancy, which is a statistical measure of the average time an individual is expected to live or survive, was estimated using equation (12) for both healthy and infected individuals. Consequently, the life expectancy for healthy individuals in the presence of HB, HIV, and TB was estimated as 28.41 years, 31.43 years, and 59.15 years, respectively. These estimates further revealed that in a population where hepatitis B is prevalent, the average life span of any healthy individual is relatively smaller as compared to higher estimates obtained for a population infected with HIV and TB, respectively. However, the life expectancy for individuals infected with HIV, HB, and TB was found to be 20 years, 25 years, and 60.37 years, respectively. It implies that the average life span of HIV patients is relatively lower than that of HB and TB patients even though they were found to be very infectious than HIV. This is because when the immune system of HIV patients gets compromised over time, it creates a way for various infections which takes advantage of the weakened immune system such as tuberculosis and other comorbidities to affect the victim.  Table 9 presents the estimates of other disease metrics (probability of infection, expected time to infection/recovery, and life expectancy for healthy/infected individuals) for HIV, TB, and HB.

### 3.9. Estimating the *P*^*n*^ Transition Probability Matrix

The *P*^*n*^ transition probability matrix was estimated from equation (14). The chain is aperiodic but not irreducible since the removed state (2) was an absorbing state. The *P*^*n*^ transition probability matrix predicts the transition probabilities for each disease at any time step.

*P* ^*n*^ transition probability matrix for HIV is estimated as

(18) $$\begin{array}{c} P_{HIV}^{n}=\left[\begin{array}{ccc} 0.93^{n} & 3\left(0.95^{n}-0.93^{n}\right) & 1-3\left(0.95^{n}\right)+2\left(0.93^{n}\right)\\ 0 & 0.95^{n} & 1-0.95^{n}\\ 0 & 0 & 1 \end{array}\right]. \end{array}$$

Clearly, *n*=1 from the estimated *P*^*n*^ transition matrix gives the actual first transition matrix *P*_*HIV*_. Therefore, the transition matrix at any time step (*n* ≥ 1) can be generated from the fitted *P*^*n*^ matrix.

Also, estimated *n*-step transition matrix for TB is given as

(19) $$\begin{array}{c} P_{\text{TB}}^{n}=\left[\begin{array}{ccc} 0.8\left(0.9831^{n}\right)+1.6\left(0.0269^{n}\right) & 0.16\left(0.9831^{n}-0.0269^{n}\right) & 1-0.9831^{n}-0.0032\left(0.0269^{n}\right)\\ 0.86\left(0.9831^{n}-0.0269^{n}\right) & 0.16\left(0.9831^{n}\right)+0.84\left(0.0269^{n}\right) & 0\\ 0 & 0 & 1 \end{array}\right]. \end{array}$$

Clearly, *n*=1 from the estimated *P*^*n*^ transition matrix (in 2 d.p.) gives the actual first transition matrix *P*_*TB*_.

Finally, the *n*-step transition probability for hepatitis B is given as

(20) $$\begin{array}{c} P_{\text{HB}}^{n}=\left[\begin{array}{ccc} 0.78^{n} & 1.17\left(0.96^{n}-0.78^{n}\right) & 1-1.17\left(0.96^{n}\right)+0.17\left(0.78^{n}\right)\\ 0 & 0.96^{n} & 1-0.96^{n}\\ 0 & 0 & 1 \end{array}\right]. \end{array}$$

Also, considering *n*=1 from the estimated *P*^*n*^ (in 2 d.p.) transition matrix gives the first transition matrix *P*_HB_.

## 4. Conclusion and Recommendation

The Markov chain model revealed that hepatitis B was more infectious over time than tuberculosis and HIV within the study population (2016 cohort at the regional hospital) although the probabilities of first infection of these diseases were relatively low. However, individuals infected with HIV had relatively lower life expectancies than those infected with TB and HB. The Markov chain model is therefore recommended as a viable technique in estimating other relevant epidemiological quantities of infectious diseases as well as generalizing the transition probabilities for future predictions.

## Figures and Tables

**Table 1:** Number of individuals at any state for cohort.

Groups	Susceptible	Infected	Dead
Susceptible individuals	*X*_00_	*X*_01_	*X*_02_
Infected individuals	*X*_10_	*X*_11_	*X*_12_

*X*_00_: number of susceptible individuals who remained susceptible at the end of the study period; *X*_01_: number of susceptible individuals who became infected at the end of the study period; *X*_02_: number of susceptible individuals who either died or remained immune after recovery at the end of the study period; *X*_10_: number of infected individuals who recovered at the end of the study period; *X*_11_: number of infected individuals who remained infected at the end of the study period; *X*_12_: number of infected individuals who either died or remained immune after recovery at the end of the study period.

**Table 2:** Number of individuals at any state for HIV at the end of the period.

Groups	Total	Susceptible state	Infected state	Removed state
Susceptible	8132	7598	502	23
Infected	502	0	478	24

**Table 3:** Number of individuals at any state for TB at the end of the period.

Groups	Total	Susceptible state	Infected state	Removed state
Susceptible	669	552	99	18
Infected	99	81	18	0

**Table 4:** Number of individuals at any state for HB at the end of the period.

Groups	Total	Susceptible state	Infected state	Removed state
Susceptible	2929	2281	623	25
Infected	623	0	600	23

**Table 5:** ML estimates of transition probabilities for HIV.

Parameters	Estimate	SE	99% conf. interval
*P*_00_	0.93	0.00283	0.923–0.937
*P*_01_	0.06	0.00263	0.053–0.067
*P*_02_	0.01	0.00110	0.007–0.013
*P*_11_	0.95	0.00973	0.925–0.975
*P*_12_	0.05	0.00973	0.025–0.075

**Table 6:** ML estimates of transition probabilities for TB.

Parameters	Estimate	SE	99% conf. interval
*P*_00_	0.83	0.01452	0.793–0.867
*P*_01_	0.15	0.01381	0.114–0.186
*P*_02_	0.02	0.00541	0.006–0.034
*P*_10_	0.82	0.03861	0.721–0.919
*P*_11_	0.18	0.03861	0.081–0.279

**Table 7:** ML estimates of transition probabilities for HB.

Parameters	Estimate	SE	99% conf. interval
*P*_00_	0.78	0.00765	0.760–0.800
*P*_01_	0.21	0.00752	0.191–0.229
*P*_02_	0.01	0.00184	0.005–0.015
*P*_11_	0.96	0.00785	0.940–0.980
*P*_12_	0.04	0.00785	0.020–0.060

**Table 8:** Estimated bootstrap confidence intervals.

Overall probability of infection	Markov chain estimate	SE	99% conf. interval
HIV	0.86	0.00385	0.84981–0.86977
Tuberculosis	0.88	0.01218	0.84753–0.91226
Hepatitis B	0.95	0.00397	0.93979–0.96005

**Table 9:** Estimates of other disease metrics for each disease.

Metrics	HIV	Tuberculosis	Hepatitis B
Overall probability of infection	0.86	0.88	0.95
Overall probability of recovery	—	1.00	—
Expected time to infection (years)	14.29	5.88	4.55
Expected time to recovery (years)	20.00^*∗*^	1.22	25.00^*∗*^
Life expectancy for healthy individuals (years)	31.43	59.15	28.41
Life expectancy for infected individuals (years)	20.00	60.37	25.00

^*∗*^Expected time to recovery from HIV and TB is also known as life expectancy for infected individuals.

## Data Availability

The Microsoft Excel Worksheet data used to support the findings of this study are available from the corresponding author upon request.
